# Reducing Drought Stress in Plants by Encapsulating Plant Growth-Promoting Bacteria with Polysaccharides

**DOI:** 10.3390/ijms222312979

**Published:** 2021-11-30

**Authors:** Roohallah Saberi Riseh, Marzieh Ebrahimi-Zarandi, Mozhgan Gholizadeh Vazvani, Yury A. Skorik

**Affiliations:** 1 Department of Plant Protection, Faculty of Agriculture, Vali-e-Asr University of Rafsanjan, Imam Khomeini Square, Rafsanjan 7718897111, Iran; r.saberi@vru.ac.ir (R.S.R.); mgholizadehvazvani@yahoo.com (M.G.V.); 2 Department of Plant Protection, Faculty of Agriculture, Shahid Bahonar University of Kerman, Kerman 7618411764, Iran; ebrahimimarzieh@gmail.com; 3 Institute of Macromolecular Compounds of the Russian Academy of Sciences, Bolshoi VO 31, 199004 St. Petersburg, Russia

**Keywords:** drought stress, plant growth-promoting bacteria, encapsulation, polysaccharides, alginate, chitosan, cellulose derivatives

## Abstract

Drought is a major abiotic stress imposed by climate change that affects crop production and soil microbial functions. Plants respond to water deficits at the morphological, biochemical, and physiological levels, and invoke different adaptation mechanisms to tolerate drought stress. Plant growth-promoting bacteria (PGPB) can help to alleviate drought stress in plants through various strategies, including phytohormone production, the solubilization of mineral nutrients, and the production of 1-aminocyclopropane-1-carboxylate deaminase and osmolytes. However, PGPB populations and functions are influenced by adverse soil factors, such as drought. Therefore, maintaining the viability and stability of PGPB applied to arid soils requires that the PGPB have to be protected by suitable coatings. The encapsulation of PGPB is one of the newest and most efficient techniques for protecting beneficial bacteria against unfavorable soil conditions. Coatings made from polysaccharides, such as sodium alginate, chitosan, starch, cellulose, and their derivatives, can absorb and retain substantial amounts of water in the interstitial sites of their structures, thereby promoting bacterial survival and better plant growth.

## 1. Introduction

Drought is a major consequence of global climate change and causes decreases in microbial functions that are essential for ecosystem sustainability and crop production. Jansson and Hofmockel [1] explored the impacts of climate change on soil microorganisms and potential ways that microbes can help to mitigate the negative consequences of climate change. Drought reduces soil organic carbon decomposition, lowers microbial biomass, and causes less CO_2_ production [2]. Drought has long-lasting impacts on the soil microbiota because it shifts vegetation to more drought-tolerant plant species and subsequently selects for root-associated microorganisms [3,4].

Santos-Medellin et al. [5] reported that long-term drought stress resulted in a sustained enhancement in growth-promoting Actinobacteria in the rice endosphere microbiome. Grassland studies have revealed a greater sensitivity to drought among soil bacteria than among fungi [6,7]. However, soil microorganisms have developed some strategies, such as osmoregulation, dormancy, reactivation, biosynthesis of extracellular enzymes, and biofilm production, that promote their survival under drought stress. Some bacteria, including Actinobacteria and Bacilli, conserve activity and become dormant under drought stress conditions to survive in drought-impacted soil [8,9].

Xerophytic plants are an essential source of drought-tolerant microorganisms. For example, 22 *Bacillus* spp. strains were isolated from the rhizosphere of guinea grass. These drought-tolerant rhizobacteria alleviated drought stress in guinea grass by the induction of proline accumulation and glutathione reductase activity [10]. Raheem et al. [11] have also isolated bacterial strains of *Bacillus*, *Enterobacter*, *Moraxella,* and *Pseudomonas* from *Acacia*, a xerophytic plant. Their studies revealed the ability of these bacterial strains to improve yields of wheat under drought stress. Plants exposed to drought stress conditions utilize three survival strategies: escape, avoidance, and tolerance. The ability of the plant to complete its life cycle before the onset of drought is termed drought escape. The escape mechanisms involve rapid plant development, the shortening of the life cycle, and self-pollination. The ability of the plant to maintain high tissue-water content, despite a reduced water content in the soil, is termed drought avoidance. Increasing water uptake from the established root system and reductions in stomatal transpiration are examples of drought-avoidance mechanisms. The ability of the plant to endure low tissue water content through adaptive traits is termed drought tolerance. Osmotic adjustment, antioxidant defense mechanisms, and increased root:shoot ratios are various mechanisms that plants utilize to tolerate the adverse effects of drought stress [12,13,14].

Association with beneficial soil bacteria is another strategy that enhances drought tolerance in plants [15]. Therefore, the direct application of plant growth-promoting bacteria (PGPB) into the soil can enhance soil properties and increase mineral fertilizer efficiency and plant nutrient acquisition. Drought is a concern that adversely affects crop yield, but it also affects the survival of beneficial microbes. Agriculturally beneficial soil microorganisms have, therefore, been encapsulated inside polymer coatings for protection against adverse environmental conditions [16,17] to improve their effectiveness in promoting plant growth under drought stress. Achieving a suitable formulation by encapsulation is a novel technology for bacterial agents, resulting in the gradual release of encapsulated bacteria into the soil, increasing the survival of bacterial agents, and thus improving their activity to reduce drought stress in plants. This subject could be a new horizon for future research. In this review article, we discuss the importance of the encapsulation of PGPB for promoting tolerance to drought stress in plants, and we summarize the current status of this research area.

## 2. Plant Responses to Drought, from Morphological to Physiological Levels

Plants perceive water deficit conditions in their roots, and molecular signals move from the roots to shoots [18]. These signals, which can include hydraulic signals, electric currents, calcium waves, reactive oxygen species (ROS), phytohormone movements, and hormone-like peptides, mediate drought stress responses in plants [19,20]. For example, an accumulation of abscisic acid (ABA) occurs in the vascular tissues of leaves in response to drought [21]. ABA promotes plant resistance to drought stress by regulating stomatal closure and inducing stress-responsive gene expression [22]. Similarly, cell elongation is inhibited under severe water deficiency [23], and drought stress reduces photoassimilation and the production of the metabolites required for cell division [24,25].

At the morphological level, lateral root growth is reduced under drought stress, whereas the primary root is not affected [26]. Another adaptive plant strategy is the generation of small roots with root hairs to provide a greater absorptive surface and thereby increase the uptake of available water. Hormonal cross-talk mediated by auxin, cytokinin, gibberellin, and ABA modulates root-system architecture under water stress [27]. The induction of enzymes related to root morphology has been reported under mild drought stress [28]. Plants also improve their tolerance to water-stress conditions by the formation of specialized tissues, such as a rhizodermis characterized by a thickened outer cell wall, a suberized exodermis, and reduced numbers of cortical layers [26,29]. Henry et al. [30] showed a decrease in the suberization and compaction of the sclerenchyma layer cells in rice plants exposed to drought stress.

Drought stress influences plants throughout the whole life cycle. The severity, duration, and timing of drought stress, and the interactions between different stresses and other factors, determine the severity of the damage experienced by drought-stressed plants [31]. At the physiological level, drought reduces plant growth and development and hampers flower production and grain filling [25]. Photosynthetic rates are reduced under drought-stress conditions mainly because of stomatal closure and metabolic impairment [32]. Chlorophyll content is strongly influenced by drought stress, with changes in activities of Rubisco and other enzymes associated with photosynthesis, resulting in oxidative damage under water deficit and the loss of photosynthetic pigment content [33,34].

Water stress also influences the acquisition of nutrients by the root and their transport to shoots. Generally, drought stress induces an increase in nitrogen, a decline in phosphorus, and no definitive effects on potassium levels [35]. Nevertheless, differences are evident in the various reports of changes in nutrient uptake under water deficit. For example, potassium uptake is decreased under water stress, as reported by Hu and Schmidhalter [36], whereas the accumulation of manganese, copper, molybdenum, zinc, calcium, potassium, and phosphorus is increased in soybean under drought stress [37].

Similar to other abiotic and biotic stresses, drought stress leads to the generation of ROS and to subsequent oxidative damage in plants [38]. Plants produce antioxidant enzymes and non-enzymatic components to protect themselves against oxidative stress. Of these, superoxide dismutase, catalase, peroxidase, ascorbate peroxidase, and glutathione reductase are the most important antioxidant enzymes, while the key non-enzymatic compounds include cysteine, ascorbic acid, carotenes, and reduced glutathione [39]. A higher antioxidant capacity was reported in drought-tolerant tomato genotypes by Shamim et al. [40].

In addition to the enhanced production of antioxidants and enzymes, plants produce osmolytes and hormones at the biochemical level to improve their tolerance against drought stress. The accumulation of osmolytes, such as glycine betaine, mannitol, trehalose, and proline, is necessary for osmoprotection and osmotic adjustment under water-deficit conditions [41,42]. Proline accumulation diminishes lipid peroxidation and ROS levels to allow the maintenance of membrane integrity [43]. The application of these compatible solutes exogenously is also effective for enhancing drought tolerance in plants [44].

Plants growing under water stress can be induced to synthesize compatible solutes by the application of selenium [45]. This mineral enhances plant growth and protective enzymatic activity levels, while reducing oxidative stress damage, increasing oxidative stress under light stress, enhancing antioxidant production to prevent senescence, and regulating the water balance of the plants for tolerance of drought stress [46]. Several studies have also demonstrated that the exogenous application of silicon can improve drought tolerance in plants [39,47,48]. For example, water-stressed wheat plants fertilized with silicon showed higher relative water contents and increased shoot dry matter, compared to unfertilized control plants under water stress [49]. Application of the phytohormone auxin also improves plant drought tolerance by regulating root development, the functioning of ABA-related genes, and ROS metabolism [50]. ABA increases drought tolerance in plants by stimulating stomatal movement, altering root architecture, regulating photosynthesis, and promoting the expression of ABA-induced genes encoding drought-related proteins [51]. Jasmonic acid is another hormone that can improve drought tolerance in plants [52].

## 3. PGPB Mitigate the Adverse Effects of Drought on Plants

The growth improvement by root-colonizing plant growth-promoting rhizobacteria (PGPR) or bacteria (PGPB) has been studied in many research scenarios [53,54,55]. PGPB play an essential role in the defense of plants against biotic pests, and the role of these microorganisms against abiotic stresses is undeniable. Water scarcity is one of the threatening environmental issues arising from climate change, and drought can reduce water availability and water quality, thereby imposing negative economic impacts, both directly and indirectly, on agriculture. Water scarcity is a severe problem and is one of the main reasons for low crop yields worldwide. Production of drought-resistant cultivars with high yields and with adaptations to different geographical areas requires long-term breeding programs and genetic engineering. Therefore, the use of beneficial bacteria with known positive roles in increasing yield and stimulating plant growth makes sense in the face of biotic and abiotic stress factors.

PGPB are viewed as a safe and ecologically complementary solution to the food security problem, along with traditional crop-breeding and genetic engineering. PGPB are associated with the rhizosphere and can improve crop productivity and plant tolerance against stresses through nitrogen fixation [56]. The mechanisms associated with induced systemic tolerance and crops with better tolerance to drought include antioxidant defenses, osmotic adjustment by accumulation of compatible solutes, production of 1-aminocyclopropane-1-carboxylate (ACC) deaminase and exopolysaccharides (EPS), phytohormone production (e.g., indole-3-acetic acid (IAA), ABA, gibberellic acid, and cytokinins), and defense strategies, such as the expression of pathogenesis-related genes [15,57,58,59,60,61]. The mechanism of plant drought tolerance induced by PGPR has been described in a recent review [62].

Bacterial strains isolated from foxtail millet in a semi-arid agroecosystem were capable of alleviating drought stress in millet by producing ACC deaminase and EPS [15]. Ghosh et al. [63] reported that drought-tolerant bacteria, such as *Pseudomonas aeruginosa*, *Bacillus endophyticus*, and *B. tequilensis,* improved drought tolerance in Arabidopsis seedlings by the secretion of phytohormones and EPS. Metabolomics analyses of *Sorghum bicolor* inoculated with rhizobacterial isolates revealed the development of systemic tolerance in plants against drought [64]. A role for EPS-producing bacterial strains for the mitigation of drought stress in wheat was demonstrated by Ilyas et al. [65], who revealed that *Azospirillium brasilense* and *B. subtilis* produced appreciable amounts of EPS and osmolytes that improved plant drought tolerance. The combination of these bacterial strains resulted in the production of higher amounts of EPS and proline (an osmolyte), and changed the levels of stress-induced phytohormones. For example, the concentration of ABA increased, whereas the concentration of other phytohormones decreased following the co-inoculation of these bacterial strains. However, seed germination, the seedling vigor index, the promptness index, and plant growth increased in response to these strains in plants under osmotic stress [65].

*Medicago truncatula* inoculated with *Sinorhizobium* sp. responded to drought stress by upregulation of translation of the jasmonic acid signaling pathway and downregulation of ethylene biosynthesis, resulting in an enhanced tolerance to drought [66]. Potato plants treated with *B. subtilis* HAS31 had higher contents of chlorophyll, soluble proteins, and total soluble sugars, and higher activities of catalase, peroxidase, and superoxide dismutase enzymes under drought stress, when compared to untreated drought-stressed control plants [67]. Table 1 summarizes some other studies on the effects of PGPB on several crops and their ability to reduce drought stress and induce systemic tolerance.

## 4. Encapsulation of PGPBs

Encapsulation tends to stabilize cells, protect against exposure to abiotic and biotic stresses, and potentially enhance bacterial cell viability and stability during the production and storage of agriculturally important strains. It also confers additional protection during rehydration [83,84]. The encapsulation of microorganisms is one of the newest and most efficient techniques to protect bacterial cells and allow for better survival in the soil after inoculation [85]. Encapsulated bacteria can be released slowly into the soil, thereby providing long-term beneficial effects on plant growth under adverse conditions [83].

Several carriers have been formulated for PGPB, with components that have included talc [86], vermiculite, perlite [87], polyacrylamide [88], carrageenan [89], sodium alginate (ALG) [90], ALG–starch [85], ALG–humic acid [91] in powder form [92,93], peats [94], liquids [95,96], and clays [97].

The encapsulation of PGPB has been used in agriculture to obtain a structure that promotes the protection, release, and functionalization of microorganisms, stabilizes the cells, protects against exposure to abiotic and biotic stresses, and potentially enhances PGPB viability and stability during the production, storage, and handling of their agriculturally utilized forms [84,98]. Table 2 shows the traditional carriers used for microbial inoculants. These carriers have several disadvantages, but the most important is their short-term effects. For example, formulations of *B. subtilis*, *P. corrugata*, and *A. brasilense* in peat or liquids have shown severe reductions in the bacterial populations [83,99], and this short-term effect has prevented any long-term impact on plant stress. Therefore, encapsulation absolutely requires the presence of a substance that is compatible with nature and that can protect bacteria from the adverse effects of stress.

Protection for PGPB must be non-toxic, preservative-free, capable of degradation in soil by microbial action, and resistant to destructive environmental factors present in the soil. Encapsulating materials must be able to maintain cell viability for different periods in the soil, preserve cell viability for three years of shelf storage, allow the progressive release of the encapsulated bacteria into the soil, be stable when stored at room temperature for extended periods, increase the number of encapsulated bacteria inoculated into the soil, and control the release of bacteria. These properties would facilitate their application to the farmer, generate an adhesive effect on seeds, and create an adequate microenvironment to preserve microbial viability and biological activity during long periods [16,83,99,101,104,105,106]. Encapsulation of beneficial PGPB has been proposed as a suitable solution to deal with drought and salinity stresses by increasing the efficiency of PGPB and reducing costs [100,107]. Schoebitz et al. [85] reported that the formulations used in the polymer mixtures for use as vehicles are essential parameters for encapsulation of PGPB to obtain successful microbial inoculants [83].

## 5. Enhancement of Drought Tolerance by Encapsulation of PGPBs

Drought stress is the primary reason for crop damage and losses, and many efforts are aimed at reducing or minimizing the effect of droughts. One promising strategy is to use nitrogen-fixing bacteria to decrease plant water use, as well as the negative environmental impact of chemical fertilizers [56]. A method is needed that can encapsulate the PGPB with a coating that will increase the efficacy and quality of the bioinoculants, while reducing the costs of application and the environmental impact [108]. Bacteria produce polysaccharides, proteins, and other biopolymers to form a protective biofilm that encourages community growth [109]. The encapsulation of bacteria within a matrix that mimics their natural environment is therefore an important strategy for protecting crops against abiotic stress. This matrix-focused strategy has already shown promise, as polymer-coated fertilizers are now confirmed to improve nutrient use efficiency [110] and to promote tolerance to salinity and drought stress.

**Figure 1:**
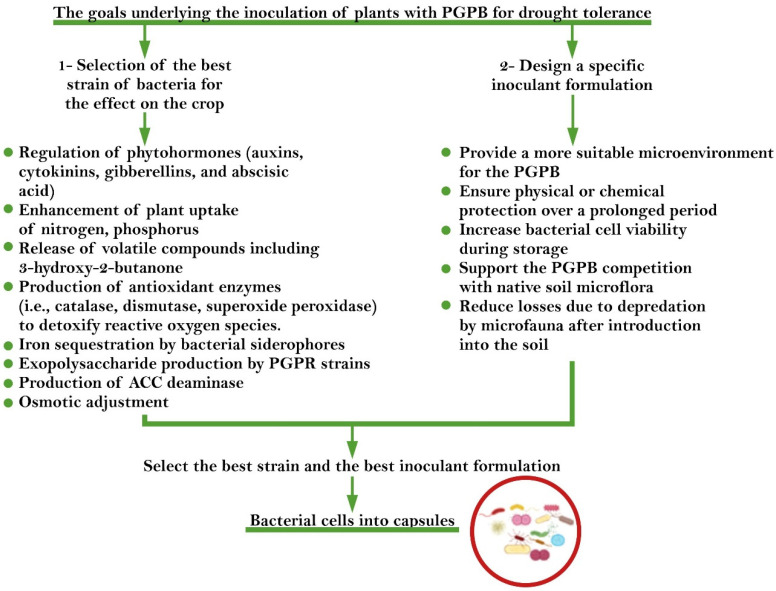
Strategies for selecting the best method and the optimal bacterial strain for encapsulation.

**Figure 2:**
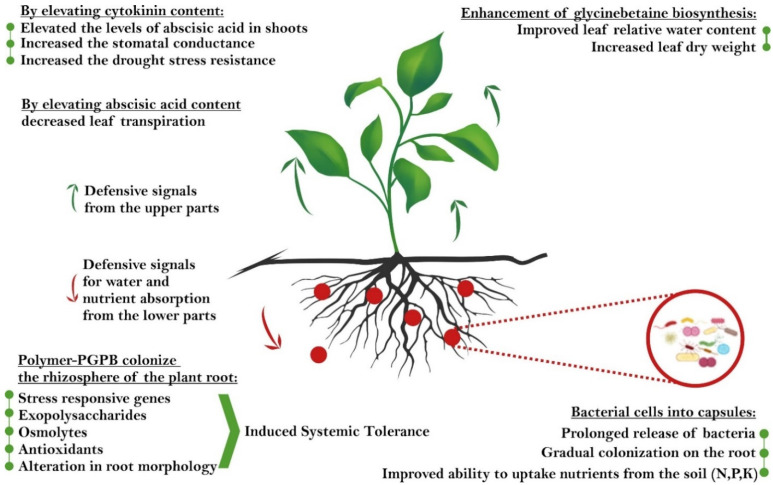
Schematic representation of the mechanism of action of polymer-PGPB soil inoculants for the protection of plants under drought stress.

Different studies have shown that PGPB populations are drastically reduced when inoculated directly into the soil under adverse (drought, salinity, and metal toxicity) conditions due to loss of their biological activity and effectiveness [111,112]. Therefore, using a protective method that traps bacteria inside a coating but that still maintains their beneficial effects under adverse conditions is a significant challenge. Many studies on encapsulation have investigated drought stress, which indicates the usefulness of this method for dehydration problems. The encapsulation of PGPB in microcapsules is a crucial method for improving cell protection and for recovering and protecting plants from abiotic stresses such as drought. Figure 1 shows the goals underlying the inoculation of plants with PGPB, while Figure 2 schematically shows the mechanism of action of polymer-PGPB soil inoculants for protection of plants under drought stress [15,62,65,101,113,114].

## 6. Polysaccharides for Encapsulation of PGPBs

**Figure 3:**
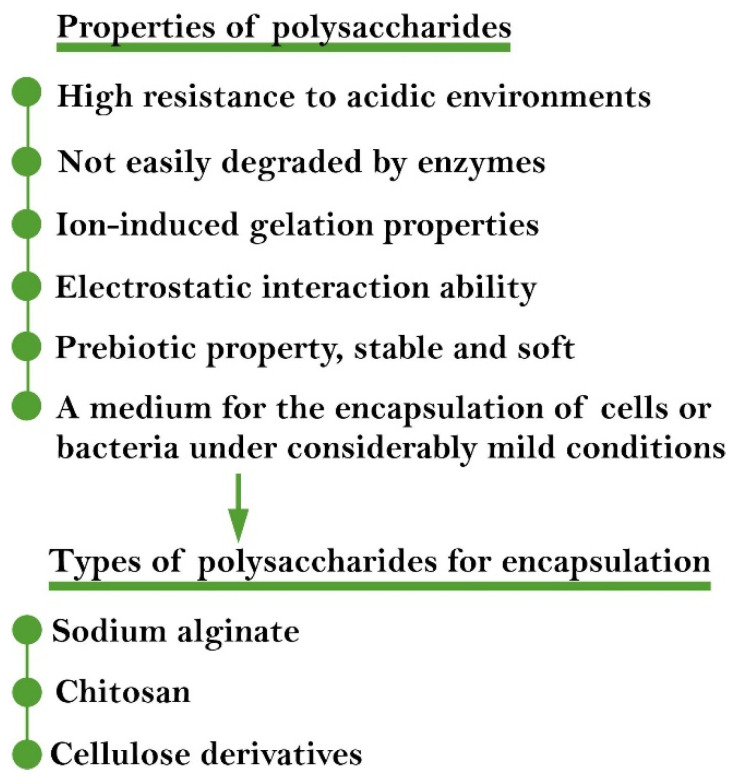
Advantages of polysaccharides for encapsulation of PGPB.

Polysaccharides are extensively used as natural capsule materials for cell encapsulation [115]. Figure 3 shows the advantages of polysaccharides over polymers [115,116] and polymeric inoculants for formulation and encapsulation [101].

The hydrogels made of polysaccharides, such as ALG, chitosan, starch, cellulose, and their derivatives, can absorb and retain an immense amount of water in the interstitial sites of their structures. The resulting polymeric hydrogels have properties of biocompatibility, biodegradability, and natural abundance, and can be widely used in medical, agricultural, and industrial applications [117]. Polymeric hydrogels have been extensively employed in agricultural systems in the past decades for the enhancement of soil density, structure, texture, water retention, and filtration rates [118]. These features come with features that favor the carrying and release of agrochemicals [119] that can improve plant resistance to drought [117,120].

### 6.1. Sodium Alginate

Sodium alginate (ALG) is a natural anionic polysaccharide obtained from brown algae and some bacteria. It consists of alternating units of α-l-guluronic acid and β-d-mannuronic acid linked by α-1,4-glycosidic bonds. ALG is widely used as a gelling agent in many biotechnological and medical processes and in agriculture. Stable hydrogels can be obtained under mild conditions by adding divalent metal cations (Ca^2+^, Sr^2+^, and Ba^2+^) to an aqueous solution of ALG. Different biologically active compounds can be trapped inside the ALG gel and then released by ALG gel degradation [121,122,123].

ALG is the most commonly used material for the encapsulation of biological control agents (PGPB) and has been extensively used to encapsulate microbial inoculants due to its simplicity of handling, viscosity, and gel-enhancing properties. Generally, ALG is safe, has a high oxygen blocking capability when dry that does not disrupt bacterial bioactivity, has no effect on the survival of bacteria even after several days of encapsulation, and is an ecologically friendly hydrophilic material. The encapsulation of bacteria in ALG beads improves cell protection and provides a prolonged release and gradual colonization of roots [56].

Successful ALG encapsulations have been reported for bacteria associated with wheat. In important crops like wheat, the factor that most limits its productivity is water availability. Drought affects the yield of wheat depending on its intensity and the phenological stage of the plant [124,125]. For example, nitrogen-fixing bacteria of the *Azotobacter* genus were isolated from the rhizosphere and used as an encapsulated inoculum to evaluate wheat growth under drought stress [56]. The isolated bacteria were screened for their nitrogenase activity and EPS production, and they were encapsulated using a sterile sodium solution. The characteristics of bead formation (encapsulation), *Azotobacter* morphology, and wheat plant growth were then evaluated. *A. chroococcum* was encapsulated in the inoculant and improved the grain yield and harvest index of the wheat under drought stress [56]. *Azotobacter*, through the colonization of the plant rhizosphere and EPS production, also alleviated the adverse effects of drought stress on wheat [56,81]. The ALG-encapsulated bacteria enhanced the activity of oxidative enzymes and improved the plant growth, physiological characteristics, and water utilization efficiency under drought stress [56].

The ability of *B. subtilis* B26 to reduce drought stress in *Brachypodium* grass involves an interaction with epigenetic variation (DNA methylation), the upregulation of different drought-response marker genes, and an increase in total soluble sugars and starch. Treatment of the drought-sensitive forage grass Timothy (*Phleum pratense* L.) with polymer-encapsulated *B. subtilis* increased plant biomass, photosynthesis, and stomatal conductance under both optimum and drought conditions. The contents of sucrose, fructans, and key amino acids (asparagine, glutamic acid, and glutamine) were also increased. A pea protein isolate–calcium alginate (PPI–ALG) matrix has been evaluated as a carrier for *B. subtilis* B26 cells for agricultural use, and the PPI–ALG microcapsules proved to be an excellent inoculation material for the release and protection of the inoculum population of bacteria in soil over a long period (112 days). The *B. subtilis* B26 cell integrity was preserved, the survival of bacterial cells was prolonged under different storage temperatures, and the release of bacterial cells from the microcapsules was detected inside the plant root and leaf tissues. The mechanism by which *B. subtilis* B26 improves plant growth under drought stress apparently involves the modification of osmolyte accumulation in the roots and shoots [126].

Another study investigated two strains of *B. subtilis* (XT13 and XT14), selected for their potential for mitigation of drought stress in guinea grass (*Megathyrsus maximus*) and maize (*Zea mays*) plants, and evaluated their effect on the stress response of guinea grass under drought. The bacterial strains were mixed with ALG to produce the formulated ALG microbeads [10] and incorporated into the soil. The dry weight of shoots and roots, the total biomass production, protein content, digestibility percentage, neutral detergent-soluble fiber percentage, ascorbate peroxidase, and proline content were all measured after 105 days. The plants under drought stress showed an increase in proline concentration and ascorbate peroxidase activity, but the co-inoculation of *Bacillus* sp. XT13 + XT14 formulated in ALG microbeads significantly enhanced the crude protein content, digestibility, and nutritional quality, while also increasing the yield of guinea grass under drought conditions [112,127,128]. The encapsulation of PGPB in microbeads positively influenced drought-stress adaptation and tolerance in guinea grass [112].

The induction of biofilm formation in *Paenibacillus lentimorbus* by ALG and calcium chloride (CaCl_2_) and its effects on drought stress were investigated in chickpea by Khan et al. [129]. The development of a biofilm is a protective strategy used by bacteria for survival in adverse conditions [130]. *P. lentimorbus* strain B-30488, with the ability to form biofilms, was isolated from cow milk under stress conditions, and this bacterium improved plant growth under non-stress and stress conditions [131]. The B-30488 strain was treated with 1% ALG and 1 mM CaCl_2_ solution, and plant seeds were submerged in the bacterial suspension until it covered the entire surface of all the seeds. The chickpea plants were harvested 120 days after sowing. During the growing period, the plants were exposed to drought conditions, with no irrigation other than one light rain event (1 mm). Several traits, such as harvest index, grain yield, and drought tolerance efficiency, were measured. RNA was extracted from the bacterial treated and untreated plants exposed to drought stress, and semi-quantitative RT-PCR was performed.

The chickpea plants inoculated with B-30488+ALG+CaCl_2_ under drought stress conditions showed an increase in shoot and root length, total chlorophyll content, and total plant biomass. The RT-PCR data analysis revealed the enhancement of dehydrin 1, lipid transfer protein, and prolyl-4-hydroxylase expression in B-30488r+ALG+CaCl_2_ treatment, compared to control plants. The ALG (1%) and CaCl_2_ (1 mM) also enhanced chemotaxis and biofilm formation of strain B-30488 under in vitro conditions. The B-30488 strain encapsulated in ALG and CaCl_2_ improved plant health and biomass yield, confirming it as a beneficial agent for drought stress amelioration in plants growing in arid areas [129]. Both ALG and CaCl_2_ are non-toxic to plants and to the environment and are useful for plant nutrition and health [132].

### 6.2. Chitosan

Chitosan is a cationic polysaccharide produced by the deacetylation of chitin, another abundant natural biopolymer. Chitosan consists of randomly distributed β-(1→4)-linked d-glucosamine and *N*-acetyl-d-glucosamine residues [133]. Chitosan has been evaluated as a potential bioinoculant carrier and can be helpful for both nutrient and mineral sequestration [134,135]. Chitosan can promote the activity of microorganisms such as PGPB, and it can induce plant responses to biotic and abiotic stresses [136,137,138]. Chitosan has bio-adhesion and cellular transfection properties [133] and can interact with PGPB. Its properties can be enhanced by combining it with other materials, making it an essential polymer for medical, agricultural, and industrial applications [139,140].

A complex of chitosan–*Methylobacterium oryzae* enhanced tomato plant growth under greenhouse conditions [141]. Chitosan nanoparticles in barley plants and pearl millet (applied by soil and foliar routes and as an emulsion) reduced the harmful effects of drought stress and increased plant growth and yield [142,143]. Plants treated with these nanoparticles showed significant increases in antioxidant defense system activity, production of phenolic compounds and osmoregulators, and crop yield [139]. Therefore, the beneficial microorganisms in these hydrogels can also be used to activate the plant’s own defense, enzymatic, and physiological systems to protect the plant from drought.

### 6.3. Other Polysaccharides

Starch combined with silicon dioxide and *Pseudomonas putida* has been used as a seed coat cover in cowpea (*Vigna unguiculata*) seeds. The seed coating containing *Pseudomonas* increased the final plant root weight, total biomass, and seed yield. Water-use efficiency (WUE) under drought stress was increased in plants grown from seeds inoculated with *P. putida*. The complex of silicon dioxide and starch with *P. putida* caused the accumulation of potassium in cowpea shoots [144]. This element is an essential nutrient for plants and plays a vital role in ameliorating drought stress and retaining cell membrane stability [144,145].

Carboxymethyl cellulose and starch form a superabsorbent material that, because of its biodegradability and stability, has been used as a hydrogel to hold irrigation water. Plants treated with these compounds continued to grow even after the cessation of irrigation [146]. Superabsorbent hydrogels have been used to manage water in the plant rhizosphere [147].

An acrylic-cellulosic superabsorbent composite containing the PGPB *Pseudomonas* (strains N33 and M25) was tested in *Eucalyptus grandis* for water-retention and protection from drought stress. The superabsorbent material served as a carrier to inoculate beneficial bacteria in the soil surrounding the eucalyptus seedlings in greenhouse conditions. This polymeric composition preserved the viability of PGPB in the soil for a long time (3 months). PGPB can stimulate plants to deploy an early response to water deficits and close stomata under drought conditions. The combination of superabsorbent material and beneficial bacteria represents an environmentally friendly system for invoking resistance to abiotic stress in plants [148].

## 7. Conclusions

Drought is one of the main abiotic factors that can severely affect the yield and quality of crops. Decreasing total yearly rainfall and increased concentration of salts in the soil are being exacerbated by climate change, making drought and salinity two critical environmental and interdependent factors with negative impacts on crop production. The production of resistant cultivars is one important strategy that can reduce crop damage caused by drought. However, the production of resistant and adaptable cultivars for different geographical areas requires long-term breeding programs.

In the rhizosphere, biological interactions occur between microorganisms and plant roots. PGPR or PGPB, such as *Pseudomonas*, *Bacillus*, and *Azotobacter,* increase the ability of plants to absorb water and nutrients and improve root growth, and play an essential role in the nutrient cycling of nitrogen, phosphorus, and potassium. These bacteria help to maintain the ecological balance of the soil and increase plant resistance to drought by affecting root morphology, plant physiological and biochemical activities, and plant growth.

Different studies have shown that PGPB populations are drastically reduced when inoculated to the soil under adverse conditions, including drought, salinity, and metal toxicity, and their biological activity and effectiveness are therefore reduced. The use of environmentally adaptive compounds, such as polysaccharide polymers, as encapsulation coatings for bacterial inocula can stabilize the bacterial cells, minimize the pressure imposed by exposure to abiotic and biotic stresses, and enhance the potential viability and stability of the bacteria during commercial production and storage as agricultural formulations. The encapsulation of PGPB is one of the newest and most-efficient techniques for protecting the cells and improving the survival of the bacteria in the soil after inoculation. PGPB can slowly penetrate from the capsules and colonize root surfaces to improve physiological and biochemical activities and the molecular signals responsible for inducing long-term resistance to drought in plants (i.e., induced systemic tolerance).

Natural polysaccharides, such as ALG, chitosan, starch, cellulose, and their derivatives, can absorb and retain immense amounts of water in the interstitial sites of their structures, which aids in bacterial survival and effectiveness. The interactions between the four critical factors of polymers, PGPB, rhizospheres, and plant roots can create drought resistance or tolerance in plants growing in arid or low rainfall areas.

## Figures and Tables

**Table 1:** Examples of PGPB strains and their mechanisms that alleviate drought stress in different plant species.

PGPB	Host	Mechanism	Reference
*Pseudomonas putida*	Chickpea (*Cicer arietinum*)	osmolyte accumulation (proline, glycine betaine) and ROS scavenging	[68]
*Bacillus* *thuringiensis*	Soybean (*Glycine max*)	modification of root structures and increased root and nodule biomass, root length, and total nitrogen content	[69]
*Pseudomonas mendocina*	Lettuce (*Lactuca sativa*)	high antioxidant enzyme activity	[70]
*Pseudomonas* *aeruginosa*	Mung bean (*Vigna radiata*)	production of ROS; increased root length, shoot length, dry weight, relative water content; and upregulation of three drought stress-genes (dehydration-responsive element-binding protein, catalase, and dehydrin).	[71]
*Burkholderia* *phytofirmans*	Wheat (*Triticum aestivum*)	improved photosynthetic rate, water-use efficiency, chlorophyll content, nitrogen, phosphorus, potassium, and protein levels in the grains of wheat	[72]
*Azospirillum* *lipoferum*	Maize (*Zea mays*)	production of phytohormones, such as ABA and gibberellins	[73]
*Bacillus thuringiensis*	Autochthonous (species *Thymus vulgaris, Santolina chamaecyparissus*, and *Lavandula dentata*)	improved the ability to uptake nutrients, and increase the shoot length	[74]
*Azospirillum sp.*	Wheat (*Triticum aestivum*)	production of plant hormones IAA, increased root growth, and formation of lateral roots, and uptake of water and nutrients	[75]
*Pseudomonas* *putida*	Soybean (*Glycine max*)	increased plant growth and production gibberellins	[76]
*Pseudomonas* *fluorescens*	Maize (*Zea mays*)	increased leaf proline, ABA, auxin, gibberellin, and cytokinin.	[77]
*Pseudomonas spp.*	Pea (*Pisum sativum*)	better grain yield	[78]
*Phyllobacterium* *brassicacearum*	*Arabidopsis thaliana*	increased biomass, ABA content, higher water-use efficiency	[79]
*Paenibacillus* *polymyxa* and *Rhizobium tropici*	Bean (*Phaseolus vulgaris*)	increased plant growth, nitrogen content, and nodulation	[80]
*Pseudomonas* *putida*	Sunflower (*Helianthus annuus*)	increased plant biomass, adhesion of soil to roots, and formation of biofilm on the roots	[81]
*Bacillus* *polymyxa*	Tomato (*Lycopersicon esculentum*)	increased relative water content, chlorophyll, protein, proline accumulation, yield	[82]

**Table 2:** Traditional carriers for microbial rhizobacteria inoculants.

Carriers	Advantages	Disadvantages	References
Peats	complex organic material with a high variability	decrease in cell concentration and adverse effects on the quality of the final product	[93,100]
Liquid inoculants	direct contact between seeds and microorganisms, increased survival of bacteria on roots	decrease in bacterial survival rates	[83,101]
Clays (as granules, suspensions, and powder)	storage for dried inoculants (large surface area, pore size distribution, and total porosity), increase the survival of rhizobia in the soil	inaccessible to predators	[83,102,103]

## Data Availability

Not applicable.
